# Some Observations Relative to the Climate and Diseases of Sierra Leone

**Published:** 1800

**Authors:** Thomas Masterman Winterbottom

**Affiliations:** of South Shields, Durham; late Physician to the Settlement at Sierra Leone.


V. *Some Observations relative to the Climate and Diseases of Sierra Leone. By* Thomas Masterman Winterbottom, *M. D. of South Shields, Durham; late Physician to the Settlement at Sierra Leone. Communicated in a Letter to* Robert Willan, *M. D. Physician in London; and by him to Dr.* Simmons.

THE Settlement of Free Town, Sierra Leone,* lies in 8° 30′ lat. N. and in long. 12° W. upon the banks of the large river Sierra Leone, or as it has been called by

* Sierra Leone has been supposed by some to receive its name from the lions with which it was infested; and by others from the tremendous roaring which the thunder makes in its mountains. Neither of these opinions however appear to be well founded; it may even admit of by old geographers, the river Tagrin, or Mitomba, about five miles from the mouth of the river. The land about the ſettlement, when viewed from the ſea, or from the oppoſite ſhore, called Bullom, appears like a number of hills, heaped one upon another, in a very irregular manner. On a nearer approach, the face of the country becomes more beautiful; the hills are covered to their very ſummits with lofty trees; and the lower grounds, which are cultivated, preſerve, by means of the heavy dews which fall, a lively verdure the whole year round, which forms a ſtriking contraſt with the darker hues of the more diſtant hills. The entrance of the river is formed by two projecting points, one on the N. W. extremity of the Bullom ſhore, improperly called Leopard's Iſland, from its reſembling an iſland when viewed at a diſtance, though it is actually connected with the main land; of doubt if a lion was ever ſeen there, as the natives have no knowledge of the animal. I am rather inclined to think it owes its name either to the diſcoverer or to ſome perſon who reſided there, and that it was called Sierra de Leon, or Leon's Mountain. This is rendered the more probable, as we find many parts of this coaſt ſtill retain the names of the firſt Portugueſe diſcoverers.

land: the other, the S. W. extremity of Sierra Leone, is a low neck of land running out into the fea, called Cape Sierra Leone, and in ancient charts, Cape Ledo, or Cape Tagrin; fometimes alfo it is called the True Cape, to diftinguifh it from a head land about fix or feven miles to the S. called the Falfe Cape, from its being frequently miftaken for the other. The diftance from Leopard's Ifland to Cape Sierra Leone, is about twelve miles; the river forms on the fouth fide feveral fine bays in its courfe from Cape Sierra Leone, which all open to the N. W.; and on the S. E. fide of one of thefe, called St. George's Bay (or Baie de France, by French Navigators) the fettlement is fituated. The breadth of the river decreafes gradually till it reaches St. George's Bay, from whence to the neareft part of the Bullom fhore, it does not exceed fix or feven miles. After paffing the fettlement, the river continues to form on its fouth fide feveral bays; at the bottom of one of which, about two miles up, called Foro Bay, is fituated Granville Town, a fettlement under the jurisdiction of the Sierra Leone Company, formed by the free blacks fent from England in 1787. The river preferves nearly the fame breadth breadth as at Free Town, for a conſiderable diſtance higher up, until about twenty miles above the ſettlement, beyond which it is navigable only by veſſels of a ſmall draught of water, it divides into two large branches, called Porto Logo and Bokell rivers, and into a ſmaller one, which from running into the Bullom country, is called the Bullom river.* The high land, from which the True Cape projects, is continued in a chain of hills which runs to the S. as far as Cape Schelling, forming part of the entrance of the great river Sherbro. From Cape Sierra Leone the hills, which are a continuation of the chain running to the ſouth, run nearly in a W. N. W. and E. S. E. direction, becoming gradually higher, to that part of them which bears about ſouth from the Iſland of Gambia, from whence they ſeem gradually to decreaſe in height. The echo which theſe hills return when a gun is fired, and the rumbling noiſe produced among them by thunder is very great, and has been noticed by all voyagers. From hence, they

* The tide of the river Sierra Leone riſes about twelve feet at ſpring tides; during the rainy ſeaſon it is very rapid, and flows about four or five miles an hour. It is high water at eight o'clock at full and change.

were called, by the Portuguefe, *montes claros.* The river, from Gambia, takes a northern direction; but it fends out a branch to the E. S. E. called Bunch River, in the mouth of which Gambia Ifland is fituated; this branch, however, does not run any great diftance, before it divides, and is loft in fmall creeks.

Gambia was formerly a flave factory occupied by the French, but now deferted; it is an ifland of confiderable extent, fituated about feven or eight miles above Free Town. The land is pretty high, but the fhore is covered with mangroves and ooze; its fituation alfo in a kind of bay, half furrounded by very high hills, renders it extremely hot, and it has always proved very unhealthy, having on all fides of it woods and fwamps. From Gambia, northwards, the river Sierra Leona becomes interfected with a number of iflands, moft of which are fmall, and many entirely overgrown with mangroves, and overflown by the tide; fome, however, are of confiderable extent, as the ifland of Robanna, upon which there is a fmall town of the natives, befides a few ftraggling houfes, built to guard their rice grounds; the land is low, fwampy, and very much infefted with mufquitoes. The iflands of Tallo and Mafabump, are the next in in point of ſize, and upon the latter are three or four towns belonging to the natives. The ſoil of Taſſo is rich, and the appearance of the whole iſland extremely picturesque, but the land is low, and the ſhore is nearly ſurrounded with impenetrable mangroves; it has beſides, ſeveral ſwamps in it of conſiderable extent, which render it very ſickly; at preſent it is almoſt uninhabited and uncultivated. To the northward of Taſſo is Bance Iſland, about eighteen miles from Free Town, upon which the Meſſrs. Andersons, of London, have eſtabliſhed a ſlave factory; this is only a ſmall and barren iſland, conſiderably elevated, and of a dry gravelly ſoil; from its ſituation, however, it is rendered extremely unhealthy: For being placed as it were in the midſt of an archipelago of low marſhy iſlands, the breeze, from whatever quarter they receive it, is impregnated with moiſture, and marſh effluvia, from the ſwamps which it paſſes over. Theſe cauſes render the air not only leſs ſalubrious, but likewiſe hotter; and the thermometer generally ſtands four or five degrees higher there than at Free Town. The inhabitants are ſupplied with water from a well upon the iſland, but the water is ſo bad, that during the rainy ſeaſon, they they prefer drinking the river water, taken when the tide is low. In the dry ſeaſon, the river is ſalt ſeveral miles above Bance Iſland.

The Bullom ſhore, ſo called from a word in the language ſignifying low land, runs in a S. E. direction from Leopard's Iſland to its eaſternmoſt point, called Tagrin point, from whence it runs almoſt north. The land, though low when compared to the high land of Sierra Leone, is pretty high when compared with the height of the coaſt in general, particularly from the river Sherbro' ſouthward as far as Cape Palmas; Cape Mount, and Cape Monſerado excepted, both which are high lands. The aſpect of the country of Bullom is extremely beautiful; the land being finely ſhaded by a variety of lofty ſpreading trees, and, in general, open and free from underwood: the ſoil is very fertile, free from ſwamps; and the ſhore is bordered by a fine ſandy beach.

The ſhore of Sierra Leone, from the Cape to within a mile or two of Gambia, is very rugged, being chiefly compoſed of rocks which abound in iron, and which lie upon a ſandy bottom. Excepting in ſome of the ſmall creeks, which proceed from the bottoms of one or two of the bays bays near the Cape, the ſhore is quite free from mangroves and ooze.

Free Town is ſeated upon a piece of ground which riſes very abruptly from the water's edge, above which it is elevated at leaſt fifty feet; from thence it riſes in a gradual and almoſt imperceptible manner, till it reaches the foot of the hills which run behind the town, leaving a ſpace of about three quarters of a mile from the bottom of the hills to the water's edge. The hills at this part are ſuppoſed to be elevated about ſix hundred feet above the ſurface of the water.*

The town is bounded on the N. W. by St. George's Bay, on the E. by another ſmall bay, and on the S. by the chain of hills above-mentioned. The ſoil is of an argillaceous

* Sierra Leone has always been noted for its fine water, which is ſuppoſed to be ſuperior to any upon the coaſt, and on that account has always been much reſorted to by ſhips. Free Town is well ſupplied from ſeveral ſmall ſprings which iſſue from crevices of the rocks. On each ſide of the town, the water runs in a conſiderable ſtream, and diſcharges itſelf into the bottom of two ſmall bays, which in the rainy ſeaſon are ſo much increaſed, as to form ſmall caſcades. The water when viewed in a glaſs is perfectly tranſparent, ſparkling, and void of ſmell or taſte.

nature nature, mixed with ſand, and here and there interſperſed with rocks; in ſome parts it is of a very deep red colour. The ſituation is very dry, incommoded by no ſwamps in its neighbourhood, and is ſcarcely troubled with muſquitoes. The town extends 2300 feet in front, and nearly the ſame in depth, though this is increaſing; it is laid out in regular ſtreets, of which nine run in a ſtraight line from the ſhore, and at right angles with it, towards the hills; theſe are interſected at right angles by four other ſtreets, running parallel to the ſhore. Each ſtreet is 80 feet in breadth, except the ſtreet next the water, which is double the breadth of the others. Every houſe in the ſtreet ſtands ſeparate, 48 feet of ground by 76 being allotted for each family to build upon. The houſes are comfortable thatched buildings, conſiſting of a ſingle ſtory, the walls of which are about 8 or 10 feet high, compoſed of wattles, unplaſtered, but commonly lined on the inſide with mats, which renders them cool and pleaſant, and preſerves a free ventilation. The floors are chiefly compoſed of clay, mixed with lime and ſand, and beat very hard upon a bottom of dry gravel or rubbiſh; which preſerves them tolerably dry.

Many

Many of the inhabitants have already built themſelves wooden houſes, with boarded floors, entirely compoſed of country wood, which is very well adapted for ſuch purpoſes; theſe houſes are raiſed two or three feet from the ground, and as they are becoming more and more common, it is probable, after another rainy ſeaſon, that this practice will generally prevail. The fire, for culinary purpoſes, is made, during the dry ſeaſon, generally without doors, but in the rainy ſeaſon it is made within on the midſt of the floor, the ſmoke finding a paſſage through the doors and windows. The ſmall ſettlement of Granville Town contains about an hundred inhabitants; the town is laid out upon the ſame plan as that of Free Town, and the houſes are built in the ſame manner: its ſituation is dry, free from the neighbouring ſwamps, and is rather more elevated than the ſcite of Free Town. This place is much infeſted during the rainy ſeaſon with muſquitoes, and as the inhabitants of Granville Town have ſcarcely ſuffered any thing from diſeaſe for two years paſt, it ſhews that thoſe inſects are not, according to Dr. Lind's opinion, to be conſidered as certain ſigns of an unhealthy country. Further, it may be remarked, that the ſettlement

 of of Port Jackfon in New South Wales, which is faid to be one of the healthieft climates in the world, is very much infefted with thefe troublefome infects

The inhabitants of Free Town amount to about twelve hundred; in general they are very induftrious, without being addicted to fpirituous liquors, and live much after the European manner.

Stock of all kinds thrives well in the colony, particularly fowls, ducks, pigs and goats. Among the wild animals met with in in the woods, the flintombo, a fpecies of antelope, is moft common; the flefh is very good and well tafted: wild hogs are alfo pretty common, and have been killed; their flefh is excellent, and preferred by many to that of the domeftic kind: buffaloes alfo have been killed, but they are not often met with; and except when they are young, the flefh is tough and not well tafted.

The river abounds with a variety of excellent fifh, which form a confiderable part of the diet of the inhabitants; oyfters are picked from the rocks and from the ftems of the mangrove trees in great numbers. Turtle are likewife very very common, the hawksbill turtle is the moſt ſo, but the green turtle is alſo frequently met with.

Rice, which is the chief vegetable production of this country, forms a large proportion of the food of the natives and of our ſettlers; it is almoſt peculiar to this rice to grow upon dry grounds, and even upon the ſides of hills; it is of a reddiſh colour when cleaned, and is eſteemed very wholeſome and nutritious. Jatropha Janipha, or ſweet caſſada, is another vegetable which grows here in great abundance; the root, when ground into flour, is often made into thin cakes by the ſettlers, which are light and palatable; like the ſweet caſſada of the Weſt Indies,* it is perfectly harmleſs, and requires no previous preparation, except boiling or roaſting, to render it fit to eat, though it is often eaten raw and taſtes much like a cheſnut: yams ſucceed very well alſo, though they were not found here in any quantity till introduced by our ſettlers. Maize or Indian corn, eddoes, plantains, ſweet

* There are two kinds of caſſada in the Weſt Indies, one called the bitter, which is noxious, and the other called the ſweet, in every reſpect reſembling that found at Sierra Leone.

potatoes, ochre, ground nuts, and various kinds of pulfe, are produced here in great abundance. Among the fruits, pine apples, oranges, limes, papayas, bananas, and a fpecies of yellow plumb, with a grateful fubacid tafte, are very plentiful. The guayaver and acajou, or cafhew, grow here, but are not plentiful; water melons have been introduced and fucceed very well; the wild vines, though extremely luxuriant, have an unpleafant acerb tafte.

In a former paper* I gave a fhort account of the weather at Sierra Leone, during the feafon in which intermittents are moft prevalent; I fhall now, from a meteorological Journal, which I kept at Sierra Leone during the whole of the year 1793, give a general view of the weather in each month. But before I do this, it may be proper to notice, though in a curfory manner, the moft ufual courfe of the feafons. The year, as in other tropical climates, may be divided with propriety into the dry and rainy feafons. The divifion into healthy and fickly feafons, which

* See Vol. VI. page 2.

holds holds in many tropical countries, as well as in many parts of Africa, cannot be obferved here, as ficknefs does not appear confined to any particular time of the year. The rainy feafon may be confidered as beginning in May, and terminating about the middle of September. The approach of the rains is ufually gradual, being ufhered in by tornadoes; they likewife decline gradually, and are carried off by tornadoes. It may be obferved that the tornadoes, which precede the rains, are, in general, lefs regular and alfo lefs frequent than thofe which carry them off.

From November to May may be called the dry feafon, though fhowers of rain do fometimes occur during thefe months independently of what falls in tornadoes. Tornadoes are fudden, and violent fqualls of wind, which almoft always come from the eaft; a few inftances, however, have occurred of tornadoes from another quarter, but thefe are very rare. The fquall feldom lafts above half an hour, and is generally attended with, or followed by fmart rain, accompanied with thunder and lightning: exceptions to this do occur, though rarely; a tornado fometimes not being accompanied with rain, and at other times with little or no thunder. The approach of a tornado is always indicated by denſe black clouds, gathering in the eaſt, which gradually increaſe until a conſiderable part of the ſky is darkened; at the ſame time, faint lightnings flaſh in the horizon, and rumbling thunder is heard at longer or ſhorter intervals. It is either calm a conſiderable time before the tornado comes on, or if there be a breeze, as the tornado comes on, it inſtantly flies round to the E.

According to a vulgar opinion, tornadoes can occur only at high or low water; a little attention, however, would ſoon have ſhewn the error of it; they come on at all times of tide, but are moſt frequent during the night, or early in the morning.

The atmoſphere at Sierra Leone is generally ſo obſcured by clouds or haze, or both, that it is very rare to ſee even part of a day attended with a clear ſky; inſomuch ſo, that it is an obſervation made by many who have been long upon the coaſt, though it more particularly applies to the coaſt to leeward of Cape Palmas, that it is a rare thing to ſee the ſun riſe in Africa. This thickneſs of the atmofphere ferves as a veil to temper the fcorching rays of the fun. The hotteft part of the day is generally about half paft two, or three in the afternoon; and the cooleft part of the day is between five and fix in the morning.

In the following TABLE is presented at one view, the highest, lowest, and medium States of the Thermometer, Hygrometer, and Barometer, during each Month, and during the whole Year. The Number of rainy Days which occurred during each Month, and in the whole Year, is likewise noted, with the Quantity of Rain which fell in each Month, and the Number of Tornadoes. The Rain attending Tornadoes is not included among the *Number* of rainy Days; but the *whole Quantity* is noted.

| Month | Thermometer | | | Hygrometer | | | Barometer | | | Rain | | Tornadoes |
|---|---|---|---|---|---|---|---|---|---|---|---|---|
| | hig. | low. | med | hig. | low. | med. | highest | lowest | medium | Days | Quant. | |
| Jan. | 89 | $74\frac{1}{2}$ | $81\frac{1}{2}$ | | | | 30,066 | 29,810 | 29,938 | 1 | 0,73 | 1 |
| Feb. | 88 | 75 | $81\frac{1}{2}$ | $59\frac{1}{2}$ | $51\frac{1}{2}$ | $55\frac{4}{8}$ | 30,018 | 29,831 | 29,924 | 3 | 0,30 | 1 |
| March | 95 | 74 | $84\frac{1}{2}$ | $55\frac{1}{2}$ | $48\frac{1}{2}$ | 52 | 29,976 | 29,888 | 29,932 | 2 | 1,12 | 4 |
| April | 95 | 74 | $84\frac{1}{2}$ | $56\frac{1}{2}$ | 50 | $53\frac{2}{8}$ | 30,016 | 29,813 | 29,914 | 3 | 1,61 | 2 |
| May | 92 | 71 | $81\frac{1}{2}$ | 61 | $46\frac{1}{2}$ | $53\frac{6}{8}$ | | | | 11 | 6,90 | 12 |
| June | 88 | 71 | $79\frac{1}{2}$ | $65\frac{1}{2}$ | 55 | $60\frac{2}{8}$ | | | | 25 | 10,16 | 2 |
| July | 85 | 73 | 79 | $69\frac{1}{2}$ | $59\frac{1}{2}$ | $64\frac{4}{8}$ | | | | 30 | 10,32 | 1 |
| August | 86 | 72 | 79 | $66\frac{1}{2}$ | 58 | $62\frac{2}{8}$ | | | | 29 | 23,14 | 0 |
| Sept. | 85 | 71 | 78 | 66 | 58 | 62 | 30,068 | 29,934 | 30,001 | 26 | 19,90 | 4 |
| Oct. | 89 | $71\frac{1}{2}$ | $80\frac{1}{4}$ | $62\frac{3}{4}$ | $52\frac{1}{2}$ | $57\frac{5}{8}$ | 30,090 | 29,980 | 30,035 | 17 | 9,08 | 15 |
| Nov. | $91\frac{1}{4}$ | $72\frac{3}{4}$ | 82 | $57\frac{1}{2}$ | 51 | $54\frac{2}{8}$ | 30,052 | 29,852 | 29,952 | 4 | 1,85 | 9 |
| Dec. | 90 | $72\frac{1}{4}$ | $81\frac{1}{8}$ | $57\frac{3}{4}$ | $46\frac{1}{4}$ | 52 | 30,060 | 29,842 | 29,951 | 3 | 1,17 | 1 |
| Whole Year. | 95 | 71 | 83 | $69\frac{1}{2}$ | $46\frac{1}{4}$ | $57\frac{7}{8}$ | 30,090 | 29,810 | 29,950 | 154 | 86,28 | 53 |

*January.*—The weather during this month, was in general clofe and fultry, efpecially during the evenings and mornings; but this was abated during the middle of the day, by the fea breeze, which commonly blew pretty frefh. The atmofphere was ufually much obfcured by haze and clouds.

The N. and E. were the moft prevailing winds. A tornado occurred on the 3d, and much heavy rain fell on the morning of the 4th. There was much thunder and lightning on the 8th.

The 8th, 11th, 12th, 16th, 28th, 29th, 30th, and 31ft days were remarkably foggy.

*February.*—The temperature of the air, though little different in abfolute heat from that of the preceding month, was rendered more agreeable to the feelings, by the frefh breezes which prevailed during the greateft part of this month.

The 1ft, 13th, 14th, 21ft, 22d, and 28th, were very foggy days. On the 13th, 22d, and 23d, there were flight fhowers. A fmart tornado occurred in the night of the 21ft. The moft prevailing winds during this month were from the N. and W.

*March.*—Notwithftanding the thermometer for the moft part ranged pretty high in this month, the temperature of the air was not unpleafant. The fea and land breezes moft commonly blew pretty frefh, and fucceeded each other with great regularity. In the mornings, however, during the interval between the blowing of the land and fea breezes, it was often clofe and fultry, though the breeze feldom fet in later than half paft eight or nine.

On the 7th a flight fhower fell. On the 8th there fell fmart rain during the night. On the 13th, 26th, 27th, and 31ft, there were tornadoes. There was thunder and lightning on the 7th, 8th, 9th, 10th, 13th, 26th, and 29th.

The 13th, 14th, 20th, 29th, 30th, and 31ft days were remarkably foggy: the only entire days in which the heat felt unpleafant, were the 18th and 19th.

*April.*—Though the range of the thermometer was pretty high this month, the heat was in general temperate and agreeable, the mornings being ufually the only part of the day which felt fultry, though this was of fhort continuance, as the fea breeze generally fprung up about nine A. M. The breeze, towards evening, evening, fometimes became lefs, or fettled in a calm, which made the air feel clofe and rather unpleafant. The atmofphere was generally hazy, and frequently obfcured with heavy clouds, as if threatening rain.

On the 4th, 20th, and 24th, a flight fhower occurred each day. A tornado occurred on the 16th, and 18th, but without being followed by thunder, lightning or rain. A fmart tornado occurred on the 23d, with thunder, lightning, and heavy rain. On the 6th, 7th, 8th, 29th, and 30th days, there was thunder and lightning.

*May.*—This month was more fultry and clofe than the preceding one, though the thermometer did not rife fo high in the prefent. The moft prevailing winds were from the W. and E. but they feldom blew frefh for any length of time. The 7th, 24th, and 29th were attended with heavy rain. On the 9th, 10th, 11th, 19th, 23d, and 25th, flight fhowers fell. Smart fhowers occurred on the 16th and 21ft.

Tornadoes appeared on the 8th, 12th, 13th, 14th, 15th, 16th, 17th, 19th, 21ft, 25th, and 26th. There were two tornadoes on the 13th; the tornado on the 19th was from the fea.

Thunder

Thunder and lightning occurred during ſome part of the 6th, 7th, 8th, 9th, 18th, 22d, 25th, 28th, 29th and 31ſt days. The atmoſphere was in general very cloudy, hazy and overcaſt.

*June.*—The temperature of the air during this month, was in general ſultry, feeling often cloſe and ſtifling, particularly when the ſun made its appearance after a ſhower of rain had fallen, and at the ſame time there had fallen little wind; though the heat indicated by the thermometer, was not ſo great as in the preceding months. In the laſt month, which might be conſidered as the forerunner of the rainy ſeaſon, there were only eleven days of rain; in the preſent month there were twenty-five, of which the 2d, 6th, 10th, 12th, 13th, 14th, 16th, and 17th days were attended with only ſlight ſhowers. On the 4th, 5th, 7th, 11th, 18th, 19th, 23d, 26th, 27th, 28th, and 29th days there fell ſmart ſhowers. On the 8th, 15th, 20th, 21ſt, 24th, and 30th days heavy rain fell. Thunder and lightning occurred during ſome part of the 1ſt, 2d, 3d, 6th, 7th, 10th, 11th, 12th, 13th, 14th, 18th, 21ſt, 24th, 26th, and 27th. A tornado occurred on the 4th, A. M. and on the 7th, P. M.

The moſt prevailing winds were from the S. and W. quarters; the breeze being in general pretty freſh, during the middle of the day, but frequently becoming calm in the mornings and evenings.

*July.*—During the whole of this month, the atmoſphere was thick and hazy, and frequently overcaſt with denſe clouds. The temperature of the air, for the moſt part, was cool, but often feeling cold with a degree of rawneſs; during the intervals of the ſhowers, however, when calm, or with only a light breeze, the air ſometimes felt ſultry and cloſe. The moſt prevailing winds were from the S. and W. and generally with a pretty freſh breeze; there were thirty days of rain in the preſent month, the 27th being the only day in which no rain fell. The 1ſt, 4th, 7th, 8th, 10th, 11th, 12th, 15th, 17th, 18th, 19th, 20th, 24th, 26th, 29th, 30th, and 31ſt, were attended with ſmart ſhowers of rain. On the 2d, 13th, 21ſt, 22d, and 23d, only ſlight ſhowers fell. On the 3d, 5th, 6th, 9th, 14th, 16th, 25th and 28th, there was heavy rain. On the 3d, 6th, 8th, 10th, and 27th, thunder and lightning occurred.

*Auguſt.*—The temperature of the air during the preſent month, was, for the moſt part cool, ſometimes

ſometimes chilly and raw. The atmoſphere was uſually obſcured by clouds and haze. The S. was the moſt prevalent wind this month, and in general it blew pretty freſh. The number of rainy days in this month was twenty-nine, of which the 8th, 14th, 22d, 23d, and 31ſt, were attended with only ſlight ſhowers. On the 1ſt, 3d, 9th, 13th, 17th, 20th, and 25th, there were ſmart ſhowers of rain. The 2d, 4th, 5th, 6th, 7th, 10th, 11th, 12th, 15th, 16th, 18th, 19th, 26th, 27th, 28th, 29th, and 30th, were days in which heavy rain fell. The 21ſt and 24th were the only days in this month free from rain, and the 21ſt was the only day which could be ſaid to be pretty clear. There were no tornadoes, nor did any thunder or lightning occur in the preſent month.

*September.*—The temperature of the air during the preſent month, was rather agreeable than remarkable for either heat or chillineſs. The atmoſphere was frequently obſcured with clouds and haze, and the tops of the hills behind the town, were covered with fog. There were twenty-ſix rainy days in this month, of which the 3d, 4th, 8th, 9th, 11th, 13th, 14th, 16th, 17th, 20th, 26th, and 28th had ſmart ſhowers. The 1ſt, 2d, 5th, 6th,

7th, 10th, 12th, 15th, 21ſt, 22d, 24th, 25th, 29th, and 30th, were attended with heavy rain. There occurred tornadoes on the 23d, 28th, and 30th. On the 1ſt, 11th, 12th, 15th, 16th, 20th, 21ſt, 22d, 26th, 27th, and 28th there was thunder and lightning, during ſome part of the day.

*October.*—The rains, which during the three preceding months, had been very ſevere, began to diminiſh conſiderably during the preſent one. The number of rainy days which occurred were only ſeventeen, of which the 3d, 4th, 5th, 13th, 14th, 16th, and 28th were attended only with ſlight ſhowers. On the 18th, 23d, 24th, 30th, and 31ſt, ſmart ſhowers of rain fell. On the 2d, 6th, 10th, and 11th heavy rain fell. Tornadoes occurred on the 3d, 4th, 7th, 8th, 9th, 14th, 17th, 18th, 19th, 22d, 23d, 25th, 26th, and 31ſt. On the 7th there were two tornadoes. On the 2d, 11th, 12th, 13th, 15th, 16th, 20th, 21ſt, 29th, and 30th, there was thunder and lightning, during ſome part of the day. The air was, in general, rendered cool and pleaſant, by a moderate breeze; but thoſe days on which the land breeze continued till near noon, it was often cloſe and ſultry during part of the afternoon, until until the fea breeze fet in, as the interval between the fea and land breezes is commonly greater at thofe times. The atmofphere was lefs gloomy than in the preceding months, though ftill hazy and often obfcured by clouds.

*November.*—The range of the thermometer was higher in the prefent than in the five laft months. The degree of moifture of the atmofphere alfo, as fhewn by the hygrometer, was lefs than in the fame months. The moft prevailing winds were from the N. and E. quarters. The heat, during the whole month, was fometimes not unpleafant, though fultry about noon, when the fea breeze fet in late. The number of rainy days in this month were only four. On the 1ft and 30th flight fhowers fell. A fmart fhower fell on the 2d, and on the 25th there was heavy rain. Thunder and lightning occurred on the 1ft, 2d, 3d, 4th, 8th, 10th, 20th, 22d, 25th, and 29th. Tornadoes occurred on the 3d, 6th, 7th, 10th, 11th, 12th, 14th, 18th and 19th.

*December.*—This month, like all the preceding, was accompanied with great hazinefs of the atmofphere, and often with low heavy clouds. The 23d was remarkably foggy; the haze haze covering the Bullom ſhore, and extending near two thirds over the river. The 8th was very cloſe and ſultry; the thermometer, at eight A. M. riſing to 85°. There were three rainy days in this month, the 2d, 27th, and 28th. A tornado occurred on the 1ſt, and faint lightnings were ſeen on the 26th and 27th.

The temperature of the air was, in general, cool and pleaſant. The winds were rather variable this month; the eaſt was moſt common in the mornings, and often continued till noon, or later. It continued to blow almoſt the whole of the 19th, 20th, and 21ſt days. About noon it uſually came from the north quarter, and towards evening veered round to weſt. The breeze was, in general, moderate and pleaſant.

Reſpecting the journal itſelf, from which theſe obſervations are extracted, it is divided into eleven columns; in which are noted, the day and hour, the height of the thermometer and barometer, the ſtate of the hygrometer, the moon's age, the prevailing winds, the appearance of the ſun, and the quantity of rain. The relative temperature of the air alſo, with reſpect to the feelings, is noticed. The obſervations were made regularly four times a day, and as nearly as was convenient, at the ſame hours. The greateſt and leaſt heights, alſo, of the thermometer, during the day, if differing much from the hour obſerved, is commonly noted.

The thermometer, conſtructed according to Fahrenheit's ſcale, was always expoſed to the free air, in a large open paſſage, perfectly ſhaded from the rays of the ſun. It was ſuſpended about ſix feet from the ground, and preſerved from the contact of ſurrounding bodies.

The height of the barometer is marked in inches and 1000 parts. This inſtrument was kept in a large airy room, elevated about ſixty feet above the ſurface of the water; the doors and windows of the room were generally kept open, but the heat of it was ſometimes increaſed by the preſence of numbers of people.

The hygrometer made uſe of was the whale-bone one invented by Monſ. de Luc, a plate of which inſtrument, with an accurate deſcription, is given in the Philoſophical Tranſactions, vol. lxxxi. page 420. The ſcale of this delicate inſtrument is divided into 100 equal parts, 0 being the point of extreme dryneſs, and 100 that of extreme extreme moiſture. There were two of theſe inſtruments alternately uſed. One being kept cloſe while the other was in uſe, they were compared from time to time, and after upwards of a year and half's uſe were not found to vary This inſtrument was contained in a box pierced with a number of ſmall holes, and ſuſpended about ten feet from the ground, in a room ſixty feet above the ſurface of the water, and in which the doors and windows were kept conſtantly open during the day. It was placed out of a current of air, and though the box which contained it was pierced with holes, yet for greater certainty, the lid was kept open about two minutes before each obſervation.

The rain gage was placed in an open piece of ground, at a conſiderable diſtance from trees, houſes, &c. upon a ſtand about four feet high. The quantity of rain which fell between any two obſervations was in general noted, except when the ſhower was very ſlight, when it was left until more had fallen.*

 The

* The figures in the column for the quantity of rain denote that the quantity of rain fallen is equal to ſo many of the diviſions of the ſcale of the rain gage in depth. The diviſion

The instruments made use of were all made by Mr. Adams, of Fleet Street, London; except the barometer, which was made by Mr. Ramsden.

On a general view of the most prevailing diseases at Sierra Leone, it appears, that remittent and intermittent fevers constitute the bulk of the complaints; and that the rest are chiefly disorders arising from a morbid increase of irritability of the constitution, or of particular parts; which, from a slight resemblance, have been usually termed febrile, though they neither arise from contagion, nor from other miasmata. The latter I shall therefore denominate diseases of erethism, in contradistinction to the truly febrile. Fevers occur indiscriminately at all times of the year, especially in persons not accustomed to the climate: They are, however, most frequent during the rainy season, and for a month or two afterwards.

division of the scale was into parts rather less than an inch; but, which, if divided by 14, gives very exactly the perpendicular height of rain upon the earth's surface.

Their

Their phenomena are nearly the ſame at all times; except in the remiſſions being more or leſs perfect; and that catarrhal ſymptoms are occaſionally ſuperadded to them.

*January.*— Intermittent fevers were the moſt prevalent diſeaſes during this month; a few quotidians occurred; but the tertian type was moſt generally obſerved, and there was not an inſtance of a quartan. Theſe diſeaſes were, for the moſt part mild, and readily yielded to a proper exhibition of bark. Convaleſcents from fevers of the preceding months, continued to recover their ſtrength, though ſlowly, and were ſtill liable to relapſes from errors of diet, or from the leaſt fatigue; and in many inſtances, from ſimple expoſure to a hot ſun. Very few remittent fevers appeared in this month, and thoſe were of the mildeſt form. Many of the convaleſcents, and thoſe who were much debilitated, though they had not been lately ſick, were liable to frequent attacks of what they termed night fevers. As this is chiefly characterized by increaſed irritability, it may be properly ranked as one of the diſeaſes of erethiſm. The patient, though weak and uncomfortable, ſeldom complains much during the day, but towards evening becomes uneafy and reftlefs; the fkin feels dry, and is rather hotter than ufual, but does not impart that burning heat ufually felt in the hot ftage of an intermittent: the principal complaint is of internal heat, or, as is ufually termed, inward fever. The pulfe is quick, and rather more frequent than natural, though fometimes it is not affected while the patient is at reft. The head is affected with pain, either on the crown or back part. The patient feldom complains of much thirft, but rather of a clamminefs of the mouth. Slight chills running down the back, fometimes ufher in a paroxyfm; but thefe are rare, and are never of any long continuance when they do occur. The other fymptoms continue the whole night, gradually becoming milder towards morning, when a partial fweat fometimes breaks out upon the head and breaft. In the morning, nothing is felt but a confiderable degree of languor and debility. Thefe fymptoms harafs the patient often for weeks and months, without appearing much to increafe the debility. They fometimes fuddenly difappear for a few days, a week, or a month, and return again. An emetic is of ufe when the ftomach is much loaded; but in general, a courfe a courſe of tonics is ſufficient. Many complained of pains in the bowels, chiefly from errors in diet, attended with ſlight diarrhœa, and ſometimes with vomiting. Slight pains ſhooting through the breaſt, increaſed upon inſpiration, were complained of by ſome. Bliſters were ſeldom employed, as the ſymptoms generally yielded to opiates, and gentle ſudorifics. Ulcers, which had been very frequent for three months paſt, were ſtill numerous; they were, in general, extenſive and foul, with a copious thin diſcharge: moſt of them had ſupervened to fevers, or ariſen from muſquitoe bites, or other trifling cauſes, in habits rendered extremely irritable. A liberal uſe of bark was generally neceſſary, and the Hydrarg. nitr. rub. applied to the ſores in ſuch proportion as to keep up a proper degree of ſtimulus, was one of the beſt dreſſings.

*February*.—Though intermittents formed the bulk of the diſeaſes of this month, they were not only leſs frequent than in the laſt, but were in general ſlight, being often brought on by irregularities, and diſappearing of themſelves in a few days. Thoſe of a more obſtinate nature chiefly occurred in perſons who had ſuffered a frequent recurrence of the diſeaſe, and neglected the proper remedies. Difeafes of erethifm ftill continued: tumefaction of the abdomen, with obfcure fluctuation in fome inftances, was very common, efpecially among children, attended with flight erethifmatic affections, refembling the train of fymptoms which takes place in what is called worm fever. The irritability in thefe cafes was fuch, that mercury fpeedily affected the mouth, when given in the fmalleft dofes; neither was its ufe attended with any fenfible good effect. A falivation, which continued a week, was produced in two children, by Calom. gr. iij, given in dofes of gr. i. Saline Purgatives, with the Decoct. Cort. Peruv. vel Anguftur. were moft effectual in promoting a cure. Œdematous fwellings of the lower extremities were the ufual confequences of long continued intermittents; but, though in many inftances, attended with evident enlargement of the fpleen, they yielded to gentle purgatives, and a courfe of corroborant remedies, ufed alternately. Two cafes of epilepfy occurred this month, both in females; in one of thefe, a girl of fixteen, who had a flight fhow of the menfes, the return of the fits was ftopped by the *Cupr. Ammon.* In the other, a woman who had borne children, children, the fits recurred every evening with as much regularity as the paroxyfm of an intermittent. The *Cupr. Ammon.* with her was tried ineffectually. The mineral folution (of Arfenic) was then exhibited for ten days, in dofes of ten drops three times a day, which put a ftop to the fits for near three weeks, though fhe often felt fome uneafinefs at the ufual period of their attack. After this time, the fits returned, though not more frequently than two or three times a week, and at uncertain times of the day. As fhe was now in an early ftate of pregnancy, and had experienced the fame complaints in former pregnancies, no more medicines were exhibited; and after the fourth month the fits entirely difappeared. An eclipfe of the moon happened on the 25th of this month, and the fame night five perfons were affected with agues; this circumftance, however, is by no means to be referred to any lunar influence, as it was an occurrence which frequently happened, and as upwards of ten times that number of convalefcents from fevers, who were ftill in a weak ftate of health, did not feel the leaft ill effects from it.

*March.*—In the prefent month, which was very healthy, fcarcely any difeafe could be faid to be more prevalent than another. Convalescents recovered their ſtrength and health very rapidly, and were not ſo ſubject to relapſes, as in the two preceding months. Intermittents had now become rare, and were almoſt all of a very mild kind. Several children were, in the courſe of this month, affected with vomiting, and ſometimes with purging. The abdomen was painful when preſſed by the hand, the pulſe was generally quick, the ſkin hot and dry, eſpecially over the forehead, and the eyes were bright. As vomiting relieved the ſymptoms, it was encouraged by a few grains of Ipecacuanha; and the bowels, if coſtive, were unloaded by a doſe of Calomel. A few drops of *Tinct. Opii. and Spt. Nitr.* generally brought out a gentle perſpiration, and carried off the complaint. A few caſes of dyſentery appeared this month, attended with ſevere tormina and teneſmus; the ſtools were bloody; the pulſe was, in general, quick and ſmall. There was great general debility and evening exacerbations. Excepting one inſtance, where a man and his wife were affected with this diſeaſe at the ſame time, there was not an example of two in a family being ſeized with it. A man, aged forty-five, rather corpulent, and much addicted to drinking of fpirits, after having, for two or three days, experienced great head-ach, giddinefs, heat and rednefs of the eyes, with fparks of light flying before them, was feized with hæmorrhage from the nofe, and loft about two pounds of blood, which relieved the fymptoms of congeftion in the head. A woman, about fifty, who had for feveral months laboured under phthifical complaints, with effufion into the cavity of the thorax, died this month.

*April.*—Difeafes from erethifm were very frequent during the prefent month; as they generally arofe in confequence of fatigue in a hot fun, and in perfons of a weakly frame of body, they were fpeedily removed by an infufion of fome bitter, affifted by a few days reft. Many complained of head-ach, which appeared to depend upon an affection of the ftomach, and was relieved by emetics. Colic pains, attended with flight diarrhœas, were alfo frequent; for the moft part, they were produced by acrid ingefta lodging in the primæ viæ, and required the ufe of evacuants. Convulfions, from dentition, occurred in feveral children this month, and in one inftance, proved fatal. Purges of calomel, opiates, and in in particular the warm bath, were attended with the moſt beneficial effects. A caſe of fever, which made its appearance in a very inſidious manner, and was accompanied in its courſe with bilious vomiting, ſpaſms and great debility, ended fatally on the fifth day. Two caſes of epiſtaxis occurred; the one, in a perſon who had marked ſymptoms of phthiſis; the other, ariſing merely from temporary congeſtion.

*May.*—The complaints of this month were few in number, and ſlight, and may be conſidered, rather as the ſequelæ of diſeaſes of former months. Four caſes of anaſarca, ſucceeding long continued intermittents, were cured by a courſe of mercury and ſquill, together with the uſe of corroborant remedies. It may be obſerved, that theſe patients bore the uſe of mercury well, a ſalivation not being ſpeedily produced. In one inſtance, the *Infuſ. Nicotian.* acted as a very powerful diuretic; but though given in ſmall doſes, it was diſcontinued, on account of the vertigo and temporary loſs of viſion which it produced. A few ſlight caſes of ophthalmia occurred, which were preſently removed by cold ſaturnine lotions. Some caſes of dyſentery appeared this month, in general ſlight; but in ſome, attended with ſymptoms of great weakneſs and irritability.

Several perſons complained of ſevere head ach, with much throbbing and pulſation of the temples, occaſioned by long expoſure to a hot ſun. The ſenſe of fulneſs of the head ſoon diſappeared after reſt, in the ſhade, leaving behind a head-ach, which yielded to gentle purgatives. In no inſtance did it reſemble a *coup de ſoleil*, a diſeaſe, which has not occurred here once in the ſpace of two years; but the ſymptoms came on gradually, in proportion to the expoſure.

Two caſes of maraſmus, in women who had been delivered of children during the laſt month, terminated fatally in the preſent one. A caſe of apoplexy terminated fatally on the fourth day, in a woman aged ſixty, of a very corpulent habit.

*June.*—Intermittents, which began to make their appearance towards the concluſion of laſt month, became prevalent during the courſe of the preſent one. Several caſes of the remittent fever likewiſe occurred; the ſymptoms were in general mild; and, in many inſtances, the exhibition of an emetic produced complete intermiſſions. Catarrhal complaints were very rife, rife, and frequently appeared as fymptoms of fever. In one cafe of fever, the catarrhal fymptoms were fevere, and appeared to confti-tute the primary difeafe; the debility, however, which occurred on the 2d day, together with very flight evening exacerbations, and a flight, but fixed pain remaining over the eyes, rendering the cafe fufpicious, the bark was liberally exhibited; and the patient recovered, though he continued weak for fome time. Slight coughs, and forenefs of throat were frequent. The cynanche parotidea occurred in a few children; the fwelling of the face was confiderable and painful; there was great reftlefsnefs, increafed heat of body, quick pulfe and fome thirft. The tumefaction of the face difappeared gradually, without being followed by fwelling of the tefticles or breafts: laxative medicines, and gentle fudorifics, were employed with advantage. Rheumatic pains became rather frequent towards the conclufion of the month, particularly in the hips and loins. Three children were feized with fevere vomiting, purging, and frequent convulfive twitches in the face and limbs, owing to their having eaten, as was faid, a quantity of raw caffada; it is, however, probable they had taken fome other other root by miftake, as no fuch inftance of its bad effects had been obferved before, although it conftitutes a confiderable part of the diet of the inhabitants: one of the children died notwithftanding the exhibition of an emetic, with plentiful dilution, the ufe of demulcent anadoyne medicines, and of the warm bath; the other two recovered by this mode of treatment.

*July*.—Remittent fevers became more and more prevalent during the courfe of this month. The onfet of the difeafe, was, in general, fudden and very fevere, few patients drooping, for any length of time, before they were confined to bed. There was great heat, naufea, and confiderable debility; the remiffions were alfo lefs evident than in the foregoing month; and the diftreffing fymptoms, as head-ach, reftleffnefs, and oppreffion of fpirits, were more obftinate. Rheumatic pains became lefs frequent during this month. Several cafes of colic occurred, which were, in general, flight. Many perfons employed in the woods in cutting timber, &c. efpecially fuch as went early in the morning, were affected with bilious vomiting, fometimes attended with purging; but thefe fymptoms foon disappeared, after the use of gentle evacuants and opiates. Dysenteries were frequent this month, but did not appear to spread by contagion; the griping and tenesmus were severe, the stools frequent, small, and chiefly composed of blood and mucus; great debility soon occurred, and all the symptoms were aggravated towards evening. After evacuating the bowels, by saline purgatives, calomel, with opium, was of great use in removing the gripes and tenesmus. The disease proved fatal in one case only, the patient being seized with it, after he had suffered greatly from fever, and had his strength much wasted.

A middle aged man, corpulent, and much addicted to drinking, was affected with singultus, without any other complaint preceding or attending it. The spasms were frequent, but not attended with pain, except a slight soreness in the region of the stomach; the hiccup was so loud as to be heard near fifty yards from the house he lived in. The pulse, during the continuance of the spasm, was raised to an hundred strokes in a minute, and was full and soft. Musk, camphor, and ol. succini, were employed, and a large blister applied in the direction of the diaphragm, without effect.

It

It yielded at length, after a continuance of four days, to large dofes of laudanum, given at every return of the complaint.

A woman, pretty far advanced in pregnancy, died fuddenly; fhe complained, a few hours before death, of a pain in her heart, (as fhe expreffed it,) but which was not fo fevere as to draw the attention of the family, and as fhe lived at a diftance in the country, no application could be made for affiftance.

*Auguft.*—Remittents continued to be the prevailing difeafes of the month. In the early part, the difeafe appeared to become lefs frequent, and the fymptoms were more mild; but the frequency of it increafed again towards the conclufion. The evening exacerbations were very fevere, and were followed by extreme debility. In addition to thefe complaints, feveral were affected with flight catarrhs, and few efcaped more or lefs of coryza; the fkin became hot and dry towards evening, with a degree of liftleffnefs and inactivity. Thefe fymptoms feldom lafted more than a few days, and required only mild *fudorifics* and *opiates*. A few patients complained this month of cephalalgia: the head-ach generally increafed in feverity towards evening, and was particularly felt acrofs the forehead, and on the fide of the head; the eyes were often red, flufhed, and unable to bear the light. The fkin was hot and dry, and the tongue white and parched. Emetics appeared to be of ufe; but, in general, fmall dofes of camphor, with opium, were moft effectual in relieving the feverity of the pain, and in bringing the paroxyfm to a termination by profufe perfpiration; the return of the complaint was effectually put a ftop to by exhibiting the bark. Blifters feldom afforded effectual or even temporary relief. A cafe of cholera morbus occurred this month, which terminated favourably.

A child, about ten years of age, died of fever, on the feventeenth day of the difeafe; her indifpofition, at firft, was fo trifling as not to be particularly noticed for fome days; though of the remittent fpecies it very much refembled the typhus of cold countries; the eyes had the fame glaffy appearance, the tongue and teeth were covered with a thick black fur, and fubfultus tendinum occurred; which fymptoms rarely appear in the fevers of this country, except the difeafe has been long protracted, and approaches proaches to a fatal termination. In this caſe, bark and cordials were employed.

*September.*—This month was more unhealthy than any of the preceding, and was felt by thoſe living on board the veſſels at a diſtance from the ſhore, as much, or rather more than by thoſe on the land. Among the black people, (who indeed, are at all times much leſs ſeverely affected with the fever than the whites,) the degree of ſickneſs, during the preſent month, was comparatively trifling, and chiefly conſiſted in intermittents of the tertian type. The whites who lived on ſhore, upwards of twenty in number, ſuffered much from ſickneſs, ſcarcely one of them eſcaping a ſevere attack of fever; many alſo had relapſes. The ſymptoms of the fever were very diſtreſſing; in particular the anxiety and reſtleſſneſs, which continued a long time after the fever had ceaſed. An intermiſſion ſcarcely ever occurred from the firſt attack; the remiſſions were obſcure, and only perceptible ſoon after the commencement of the diſeaſe. An emetic was generally exhibited immediately on the commencement of the complaint; and as ſoon as the ſtomach could bear it, the bark, with opium occaſionally, was exhibited in large doſes.—

Three patients died this month of the fever, on the 9th and 10th days of the diſeaſe. Inſtances occurred of ſevere vomiting, gripes, and purging, from eating caſſada which had ſtood in a braſs veſſel, in a woman, and a child about ſeven years old. The woman was immediately affected with ſevere vomiting and purging, which, though diſtreſſing for a time, carried off the irritating cauſe; the child having vomited only once or twice, was more ſeverely affected. The pulſe was ſmall and quick, the extremities were cold, the eyes much diſtorted; and the patient appeared to be in much pain. By means of an emetic, which operated both upwards and downwards, theſe ſymptoms diſappeared, except ſlight convulſive twitches of the face and limbs, and ſtarting during ſleep, which continued for a day or two, and then went off. Two caſes of cholera morbus appeared; in one man it was attended with very ſevere ſpaſms of the legs, and ſeemed to have originated from expoſure to a current of cold air when he was much heated by having worked in a hot ſun. In the other caſe, the vomiting was chiefly ſevere, and happened after working all day in the woods, and ſpending the night there. Both the patients recoverd by ſmall doſes of opium, fomentations, &c. A caſe of gaſtrodynia which occurred was cured by opium and the ſpir. æther. nitr.

*October.*—From the commencement of the preſent month the remittent fever gradually became leſs frequent, and towards the conclusion, intermittents were very general. Caſes of ophthalmia were alſo frequent: they appeared to ariſe from cold, and were always attended with conſiderable effuſion of tears, and inability to bear the light. In one inſtance the pain was ſo ſevere as to require large doſes of opium to moderate it, and a ſtrict uſe of the antiphlogiſtic regimen, except bleeding. Cold ſaturnine lotions with opium, applied externally, were very uſeful in abating pain, and relieved the turgid ſtate of the veſſels. Towards the end of the month, pains of the bowels, reſembling colic pains, became frequent; and in ſeveral inſtances the conſtipation was ſo obſtinate, as to require repeated doſes of calomel, ol. ricini, and ſal. cathart. Fomentations to the abdomen afforded ſpeedy but only temporary relief.

A woman, after having been employed all day in waſhing while expoſed to a hot ſun, was feized, late in the night, with very fevere vomiting and purging, acute fixed pain in the bowels, and frequent cramps of her legs; by the ufe of mild diluents, fomentations to the abdomen, and fmall dofes of tinct. opii, given at fhort intervals, the fymptoms abated; but they returned the next night about the fame time, with equal violence. The fame plan of treatment was purfued with advantage, and a few dofes of bark were given; after which there was no return of the fymptoms.

*November.*—The complaint of the bowels, which made its appearance towards the end of laft month, became fo frequent during the prefent one as to refemble an epidemic. The natives, in different parts of the river, did not efcape it, and fo frequent was it among them, that according to their cuftom, they attributed it to the effects of witchcraft. The pain was, in general, confined to the left fide, between the umbilicus and fpinous procefs of the ilium; but fhooting pains were fometimes felt in the epigaftrium, with a fenfe of tightnefs: the abdomen, when preffed, was ufually fore. The pain was not conftant, but generally abated for a few minutes, and returned with greater violence. Sometimes there was a fufpenfion fuſpenſion of pain for a few hours, but an exacerbation conſtantly happened about evening. The pulſe was always ſoft, and, except during the ſeverity of the pain, when it increaſed in quickneſs, was not much affected. The tongue was white and furred, and much thirſt attended. In many inſtances, where the pain was on one ſide, a conſiderable degree of pulſation could be felt from the meſenteric arteries. The conſtipation was as obſtinate, and the other ſymptoms were more ſevere, in this than the laſt month. Sudorifics and warm cordial medicines produced no good effect. Large doſes of opium afforded only temporary ſuſpenſion of the pain. The frequent application of fomentations to the abdomen, not only mitigated the pain, but often procured a little ſleep. The moſt certain relief was produced by an injection of turpentine. In one caſe, where the patient had been ill three days, after a drachm of camphor, and two drachms of tinct. opii. had been taken during the night, with little more effect than that of rendering the pain more obtuſe; an enema of turpentine was followed by immediate eaſe, and produced ſeveral ſtools, which large doſes of ol. ricini had failed in procuring. In another inſtance, where the patient had for four days fcarcely obtained any relief from pain, though every means had been ufed except injections, calomel, united with opium, was given in fmall dofes, night and morning. A falivation was produced after eight grains of calomel had been taken, which continued copious for ten days, and then gradually ceafed. The patient felt no remains of pain from the time his mouth was affected, and foon after the falivation abated, recovered his health and ftrength. A few cafes of intermittents occurred this month, which were in general flight.

A young man, without having any previous indifpofition, was, while fitting in a chair, feized with profufe hæmorrhage from the nofe, by which near two pounds of blood were loft, and fainting at length put a ftop to the hæmorrhage; the debility induced was very great, but was removed by bark joined with acids. A cafe of remittent fever terminated fatally this month, on the fourth day of the difeafe, being towards its conclufion attended with fevere vomiting, and fpafms all over the body.

*December.*—This month was extremely healthy, more fo than any of the preceding. A fingle cafe of remittent fever occurred this month, month, in a woman, who had been delivered about ten days, after an eafy labour. It was not accompanied by any affection of the bowels, but terminated fatally on the fixth day after the attack, owing in great meafure to her obftinacy, in refufing medicines till it was too late. Except the cafe of fever, and one of peripneumony in a woman, nearly at the term of parturition, no acute difeafe of any confequence occurred in the prefent month. The peripneumony began in the former month, but was not regarded by the patient until attended with confiderable dyfpnœa, hectic flufhings, and night fweats ; the pain was very acute under the left breaft, and there was a confiderable expectoration of a puriform matter, which had been preceded by frequent flight chills ; the pulfe was fmall, but hard and quick ; a blifter had been applied to the pained part, but without effect. An opiate was exhibited every night, and an emetic compofed of antim. tart. and vitriol. cupr. aa gr. ii. was given every fecond morning, and during the intermediate days, the pilula fcillæ *Pharmac. Lond.* was ufed. By the ufe of thefe remedies the fymptoms gradually abated, and at the end of this month no other complaint remained but a flight degree of hoarfenefs. Early in in January ſhe was delivered of a healthy but ſmall child, at the full period, which ſhe has ſuckled for two months paſt without inconvenience.

A perſon, who had long laboured under aſthmatic complaints in America, but during a reſidence of near two years in Sierra Leone had never experienced any ſevere attack, had, during the preſent month, three ſeveral attacks of dyſpnœa approaching to orthopnœa, but which was each time ſpeedily relieved by full vomiting. There occurred two caſes of a periodic nervous head-ach, attended with great irritability, which were cured by a courſe of tonic remedies.

I have thus given a conciſe view of the diſeaſes that prevailed at Sierra Leone, in the courſe of one year; and I ſhall conclude it with obſerving that while other countries have been gradually improving in cultivation, and thereby changing even the nature of the climate from unhealthy to healthy; Africa, depreſſed by the trade in its inhabitants, remains in the ſame rude, uncultivated ſtate, in which it was centuries ago. It has proved the grave of many Europeans, but, a great or perhaps, the the chief part of this mortality may be attributed to causes independent of the general insalubrity of climate. Of the Europeans who have died in Africa, the greater part are either seamen or traders. The former, are in every country remarkable for imprudence and carelessness of their health; and it has been observed, even in temperate climates, that they are generally short lived. But in Africa they labour under disadvantages unknown elsewhere, and which, of themselves might fully account for the extraordinary mortality. On board of Guinea ships, there is often no regular allowance of liquor, and as the seamen employed in the African trade, are commonly dissolute, they eagerly seize every opportunity, while laying on the coast, of procuring spirits, even selling their wearing apparel and their beds, if, as generally happens, the captain refuses them any other means of purchasing them. In this way, few are possessed of clothes to shift themselves, when drenched with the heavy rains which fall in the wet season, or of any other bed than the bare deck, or a cable, whereon to sleep at night. During the day, they are often exposed to row in boats, under a scorching sun, and when fatigued tigued with this laborious employment, frequently fall aſleep in the boat, expoſed to its ardent rays ; this extreme of heat is commonly followed by expoſure to the heavy chilling dews, which begin to fall after ſun-ſet, and which affect their bodies the more readily, as they have been heated by the labours of the day ; this is a very frequent cauſe of the obſtinate rheumatic pains with which many in theſe ſituations are affected.

Theſe cauſes of diſeaſe, it may be ſaid, the African has in common with the Weſt Indian trade ; this muſt be partly granted, but the following evils are peculiar to the former. The ſtation of veſſels trading to the coaſt is often ſuch, that it may be conſidered as the focus of diſeaſe. Veſſels frequently remain for many months on the coaſt, perhaps in rivers, near an oozy ſhore, covered with lofty mangroves, which exclude the cool ſea breeze, permitting them only to receive, during the night and part of the morning, a peſtiferous breeze from the land, impregnated with moiſture, and the exhalations of marſhes. During their ſtay here, the men are perhaps ſent up creeks in open boats, in which they continue for ſeveral ſucceſſive days, expoſed to all the viciſſitudes of of heat and cold. The unbounded licence of a flave fhip, expofes the men likewife to all thofe evils which arife from excefs of venery; while the influence of this trade on the minds of their fuperior officers, often expofes them to the ftill more fatal effects of fcanty diet, and of furious and ungovernable paffions.

The conduct of thofe Europeans who form little factories upon the coaft for trade, is feldom much better than that of the unthinking failor; the moft part of thefe traders have been originally common failors, who from long refidence upon the coaft, have become acquainted with the trade. Their mode of life is generally extravagant, and licentious; they adopt readily all the cuftoms of the natives, fo that it has become a proverb among them, "that a white man foon turns black." The miferable houfes, or rather hovels, they inhabit, often built of mud, without any flooring but the damp earth, and much inferior to thofe of the natives, fhow how little they avail themfelves of European arts.

Their places of refidence are chofen without any regard to health, but according as they may be advantageoufly fituated for trade; for which reafon, they are generally on the banks of ſome river, or up ſome ſmall winding creek. The towns of the natives are uſually ſurrounded by trees, &c. ſhut up from receiving the cooling breeze ; a ſituation preferred by them, on account of the ſecurity it gives from ſudden attacks, although its baneful effects are felt by themſelves, and proved, by the ſickneſs, which every rainy ſeaſon prevails among them, and by the feeble emaciated bodies of their old men. That health may be retained in Africa, as well as in other parts of the world, when attention is paid to ſituation, &c. appears proved by the inſtance of Free Town. The ſcite of this town is ſuch as to avoid the inconveniencies above mentioned, and the ſalutary conſequences of this, have ſhewn themſelves by the very trifling degree in which the ill effects of the laſt rainy ſeaſon have been felt by the inhabitants.

